# RNAi in the regulation of mammalian viral infections

**DOI:** 10.1186/1741-7007-10-58

**Published:** 2012-06-26

**Authors:** Kuan-Teh Jeang

**Affiliations:** 1 The National Institutes of Health, Bethesda, MD 20892-0460, USA

## Abstract

Although RNA interference (RNAi) is known to play an important part in defense against viruses of invertebrates, its contribution to mammalian anti-viral defense has been a matter of dispute. This is surprising because all components of the RNAi machinery necessary for robust RNAi-mediated restriction of viruses are conserved in mammals, and the introduction of synthetic small interfering RNAs (siRNAs) into cells efficiently silences the replication of viruses that contain siRNA complementary sequences in those cells. Here, I discuss the reasons for the dispute, and review the evidence that RNAi is a part of the physiological defense of mammalian cells against viral infections.

## 

RNA interference (RNAi) is a process in which small non-coding RNAs (of endogenous or exogenous origin) are incorporated into a multi-protein RNA-induced silencing complex (RISC) in cells to silence the expression of a sequence-homologous target RNA [1]. Three major types of small non-coding RNAs function as RNAi: the piRNAs (PIWI-interacting RNAs), miRNAs (microRNAs), and siRNAs (small interfering RNAs). piRNAs and miRNAs are endogenous, small non-coding RNAs transcribed from cellular loci and then processed to generate fragments that engage with the downstream silencing machinery. Until recently, siRNAs were thought to be exclusively processed from the exogenous RNA of pathogens (for example, viruses) that infect the cell, but that view changed with the discovery of abundantly expressed endogenous siRNAs (endo-siRNAs) in animal cells [2,3]. Currently, mammals are known to have hundreds of thousands of different piRNAs, produced from gene clusters of repetitive elements, and more than 1,000 different miRNAs; the number of endo-siRNAs still needs to be fully clarified.

Simplified representations of the different RISC complexes are shown schematically in Figure 1. miRNA biogenesis requires the RNAse III proteins Drosha and Dicer, while siRNA processing depends solely on Dicer, and the nuclease(s) required for piRNA processing remain(s) unidentified [4]. Short double-stranded RNAs (dsRNAs) are bound to form the miRNA- and siRNA-RISC complex while the biogenesis of the piRNA-RISC can arise from either a single-stranded precursor RNA or through a 'ping-pong' mechanism [1]. A major constituent of the miRNA-RISC and siRNA-RISC complexes is the AGO protein; the parallel constituent in piRNA-RISC is the PIWI protein. In the RISC complex, a guide RNA strand is retained that captures target mRNA through complete or incomplete sequence complementarity. The RISC complex then may either inhibit translation of the mRNA or, through the so-called slicer activity of the AGO and PIWI proteins, degrade it, thus silencing the gene from which it was transcribed.

**Figure 1:**
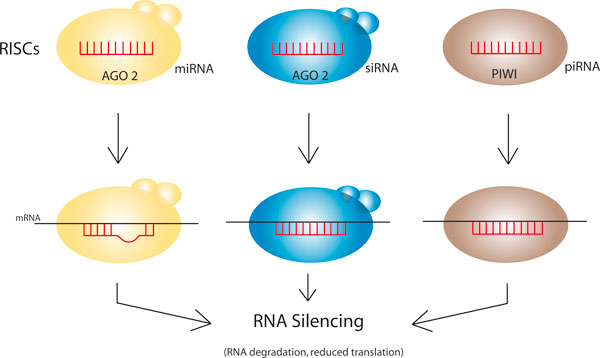
**miRNA-, siRNA-, piRNA-RISC complexes effect complementarity-driven silencing of targeted mRNAs**. Small miRNAs, siRNAs, or piRNAs (red) serve as guide sequences within the RNA-induced silencing complex (RISC) to capture target mRNA via incomplete (miRNA) or complete (siRNA, piRNA) base-pairing. The expression of the targeted mRNA is silenced either by RNA degradation or by inhibition of translation.

One of the earliest descriptions of RNAi was in *Caenorhabditis elegans*. Early on, it was found that mutations affecting RNAi function in *C. elegans *and *Drosophila melanogaster *produced apparently normal organisms, but that these mutations increased the susceptibility of mutated animals to infection by viruses [5,6]. These findings suggested an evolutionary role for RNAi in the defense of cells against pathogenic viral infections. Indeed, this rationale is consistent with the role of RNAi in post-transcriptional gene silencing of plant viruses [7] and with the conservation of an RNAi-like antiviral defense mechanism using small CRISPR (clusters of regularly interspaced short palindromic repeats) RNAs in prokaryotes [8]. The need for conservation of an analogous antiviral system in vertebrates, however, has been questioned on the grounds of their advanced adaptive immunity to viral (and non-viral) pathogens and on the emergence of an interferon-based defense mechanism. Silencing mechanisms that depend on RNAi are, however, already known to operate in vertebrates to protect the germline DNA from transposons and endogenous retroviruses; and as discussed below, they appear also to operate in somatic cells.

##  RNAi and regulation of retroelements and viruses

Non-virologists are often surprised to learn that nearly 50% of the human genome is made up of virus-like transposable elements (TEs) [9], which are composed mostly of retrotransposons replicated through reverse transcription, and DNA transposons propagated through a cut-and-paste mechanism. Included among the TEs in the mouse and human genomes are distinctly recognizable endogenous retroviruses (ERVs; integrated retrovirus sequences that have entered the germline), with 5 to 8% of human DNA estimated to consist of human endogenous retrovirus (HERV) elements that segregate into 26 phylogenetically distinct retroviral lineages [10]. These ERVs are likely to be fossilized remnants of anciently endogenized virus infections. While active human ERVs are rare [11], human non-ERV TEs (for example, short interspersed elements - SINES - and long interspersed elements - LINES) remain active for transposition, accounting for 1 new insertion every 100 to 200 human births or roughly 1 in every 1,000 human genetic mutations [12]. By contrast, mouse ERVs are numerous and highly active and cause approximately 10% of spontaneous mutations in inbred mice [13].

Active replication of ERVs and virus-like elements needs to be suppressed in the germ line because they cause novel deleterious germline mutations. In mouse germ cells, piRNA-mediated silencing has been shown to be important for repressing TE activity [14]. However, ERV and retrotransposon activities are not limited to the germline; they also occur in somatic cells where they can induce disease, in particular ERV- and retrotransposition-associated cancers [15,16]. This raises the question of whether mechanisms also exist to protect somatic tissues. There is evidence for two such mechanisms. First, somatic cell endo-siRNAs, as recently described, may act to control ERV and TE activity [3]. Second, emerging data have unexpectedly revealed that piRNAs are not confined to germline cells, but are also abundant in the somatic tissues of fruitfly, mouse, and rhesus macaques [17], in the neurons of *Aplysia *[18], and in a human T cell line [19]. PIWI mRNA and MIWI protein have also been detected in macaque and mouse somatic tissues [17]. If these new discoveries are confirmed, then ERV/TE-suppression in somatic cells may be mediated by piRNA-RISC.

There is also direct evidence supporting a role for RNAi in regulating viral infections in mammalian cells. As in the early studies that showed RNAi pathway mutations in *C. elegans *and *D. melanogaster *increased these organisms' susceptibility to infection by viruses [5,6], mutations in or perturbation of RNAi pathway components in mouse [20], monkey cells [21] or human cells [22,23] increase the replication of vesicular stomatitis virus (VSV), influenza A virus, and human immunodeficiency virus (HIV-1), respectively.

##  miRNA-mediated regulation of viral infection

The human genome encodes more than 1,000 different miRNAs; these miRNAs and their miRNA-RISC complexes recognize RNA targets through imperfect base-pairing [1]. *In silico *analyses based on complementarity of miRNAs and their putative mRNA targets have led to estimates that miRNAs may regulate up to 30% of protein-coding human mRNAs. Not surprisingly, early analyses of the more than 1,000 human miRNA sequences aligned against a large dataset of pathogenic mammalian viral genomes indicated that most, if not all, viruses are recognized by one or more cellular miRNAs [24].

**Figure 2:**
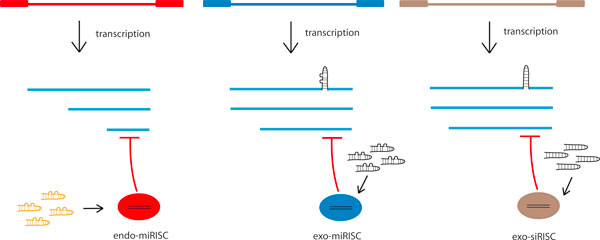
**Several ways that RNAi can regulate viruses in mammalian cells**. Left: cell-endogenous endo-miRNAs are engaged in RISC complexes to target partially homologous viral transcripts. Middle: virus-encoded miRNA can be processed as exo-miRNAs that are engaged with RISC for interaction with other viral RNAs. Right: viruses that contain shRNA sequences (for example, lentiviral shRNA libraries) are processed into exo-siRNAs. The double-stranded RNAs (hairpins) are processed in the RISC into single-stranded guide RNAs that bind to complementary sequences in the mRNA, thus recruiting RISC, which degrades the mRNA or inhibits its translation. Transposable elements and endogenous retroviruses produce endo-siRNAs in mammalian cells. Both exo-siRNAs and endo-siRNAs can be incorporated into RISC complexes in mammalian cells to silence homologous target RNAs. Multiple transcripts (blue) are indicative of differently spliced RNAs.

Numerous studies now report the direct regulation of mammalian viruses by host miRNAs (Figure 2). Thus, human liver-specific miR-122 has been shown to functionally augment hepatitis C virus (HCV) replication [25], while more than half a dozen human miRNAs, including miR-199a-3p, miR-210, and miR-125a-5p, are found to repress hepatitis B virus (HBV) replication [26]. Other examples include miR-323, miR-491, and miR-654 targeting influenza virus, miR-27 and miR-93 targeting VSV, and miR-28, miR29a, miR-125b, miR-150, miR-223, and miR-382 targeting HIV-1 [27]. More recent data suggest that herpes viruses (for example, Epstein Barr virus (EBV) and Kaposi's sarcoma herpes virus (KSHV)) are targeted by several cellular miRNAs, including the miR-17/92 and miR-106b/25 clusters [28-30], coxsackie virus is targeted by miR-342-5p [31], and human papilloma virus (HPV) is targeted by several cellular miRNAs [32]. The list of cellular miRNAs implicated in regulating mammalian viruses promises to grow much longer. Indeed, in a survey of more than 25,000 individual HCV, HIV-1, HPV and HBV sequences, it was found that there is strong conservation and preservation of cellular miRNA-targeted sites within those viruses, prompting the authors to conclude that 'human microRNAs effectively contribute to the host defense by targeting essential viral genes, thereby reducing the replication efficiency of the virus' [33]. Taken together, the accumulated findings support the concept that ambient miRNAs expressed in host cells represent a first layer of bioactive encounters that form a part of the cell's overall antiviral arsenal.

##  Efficient processing of viral short hairpin RNAs and miRNAs occurs in mammalian cells

Despite the above findings, there remains some contention about the physiological function of RNAi in regulating viruses in mammalian cells. This contention may be caused in part by expectations of mammalian cells/mammalian viruses based on results from invertebrate cells/invertebrate viruses. For instance, when invertebrate viruses infect mosquito cells, the incoming viral dsRNAs or replication intermediates are frequently processed into small exogenous siRNAs (exo-siRNAs) [34]. By contrast, when mammalian viruses, such as HIV-1, infect human cells, the production of processed viral exo-siRNAs is rare [19,35]. Some have interpreted these results to mean that invertebrate cells sense and process viral dsRNAs and then deploy exo-siRNAs in antiviral RISC complexes while vertebrate (mammalian) cells cannot perform these functions. This interpretation however is challenged by experiments in which an authentic short hairpin RNA (shRNA) was engineered into the HIV-1 genome and the genome introduced into human cells, with ensuing efficient siRNA production from the 'viral-shRNA' [36]. Moreover, a version of the above experiment is performed hundreds, if not thousands, of times every day by investigators who use shRNA libraries cloned into lentivirus vectors to transduce human cells for the purpose of silencing specific target genes. In every instance, the lentivirus-shRNA is recognized and processed by human cells faithfully into the expected siRNA (Figure 2).

How then does one reconcile the above observations? One possibility is that human (mammalian) cells, rather than being unable to efficiently recognize and process viral dsRNAs or shRNAs into siRNAs, may actually be more proficient than invertebrate cells at processing and using siRNAs in antiviral RISC complexes against invading viruses. In this case, RNA viruses with double-stranded shRNA-like sequences amenable for processing into siRNAs might be subject to more potent negative selection in human than in invertebrate cells. Over time, strong stringent selection in mammalian cells (and relaxed selection in invertebrate cells) would result in many human viruses being devoid of shRNA-like or dsRNA sequences, while the less robustly restricted invertebrate viruses would still keep shRNA and/or dsRNA sequences. Hence, today's human cells would face many viruses largely lacking shRNA/dsRNA sequences, accounting, in part, for the rarity with which viral exo-siRNAs are detected in mammalian infections (see, for example, [37]). On the other hand, invertebrate cells would still encounter many viruses that harbor shRNA sequences, and consequently viral exo-siRNAs would more frequently be found in infected invertebrate cells [34]. In short, the argument is that human cells do, in fact, efficiently process viral exo-siRNA and can use siRNA-RISC as an antiviral defense, but the rarity of this process in human cells may be because many mammalian viruses have already been tightly selected by human cells not to maintain siRNA-producing sequences.

They do, however, encode miRNA sequences [38,39]. These viral miRNAs are processed efficiently in human cells, are engaged in miRNA-RISCs, and are frequently used by the cell (or the virus) to target other viral transcripts. Examples of this type of usage include the human cytomegalovirus (CMV) miR-112-1 targeting the CMV IE1 viral RNA [40], the EBV BART miRNAs targeting the EBV LMP mRNAs [41,42], and the HSV miR-H2, miR-H3, and miR-H4 targeting viral ICP0 and ICP34.5 mRNAs [43]. Similar miRNA-mRNA targeting can also occur in the case of a model retroviral infection [44]. Taken together, these findings support the view that mammalian cells employ viral exo-miRNA-RISCs to regulate viral infections, paralleling invertebrate and plant cells that use viral exo-siRNA-RISCs to regulate their cognate viruses (Figure 2).

## Outstanding questions and future perspectives

The above arguments apply to retroviruses and herpes viruses, which have been extensively studied for RNAi generation. What remains unaddressed is the processing of the many plus-sense, minus-sense, and human dsRNA viruses. These viruses present fully dsRNA substrates as part of their genomes or as replication intermediates. To date, they have not been extensively investigated for the biogenesis of viral non-coding RNAs, and future findings from these viruses could yield important insights.

Several aspects of the RNAi-virus-host cell interaction also merit closer scrutiny. One issue is whether the expression of endo-siRNAs in mammals is similar between germline tissues and somatic tissues. The data for somatic tissues are currently incomplete. The emerging finding that piRNAs are present in both germ and somatic cells raises the possibility that endo-siRNAs conform to an analogous pattern.

A second issue is the dynamic strike-counterstrike interplay between cells in which RNAi serves to combat viruses and viruses evade RNAi to successfully replicate in cells [45]. One view is that efficiently replicating viruses must encode RNAi suppressors. Indeed, while many mammalian viruses do apparently have RNAi suppressor moieties [27], an RNAi suppressor function is only one of several means (for example, shielding of the virus genome from RNAi, sequence changes in the viral genome to evade RNAi, virus modulation of cellular miRNA expression, and virus adaptation to cellular RNAi) [38] at the virus' disposal to skirt cellular RNAi restriction. Indeed, shielding of the viral RNA genome from RNAi [46], changes in viral sequences to evade RNAi [47], and virus-modulation of cellular miRNA-expression [48-50] have all been reported for HIV-1. Viruses such as HIV-1 that are highly mutable may evade RNAi efficiently through target sequence changes; these viruses do not need a strong RNAi suppressor [37,51,52], if at all [53]. On the other hand, less mutable viruses may require strong RNAi suppressors to mitigate RNAi restriction in order to replicate optimally.

A final issue is the increasingly convincing new evidence for the existence of natural antisense transcripts in human T-lymphotropic virus (HTLV)-1 and HIV-1 [54-58]. Inside cells, these antisense viral RNAs can, in principle, form long RNA duplexes with their complementary sense transcripts. The fate of these dsRNAs and what new functions they may provide promise to keep virologists busy for the next several years.
